# Using a mixed method to identify communication skills training priorities for Chinese general practitioners in diabetes care

**DOI:** 10.1186/s12875-022-01868-8

**Published:** 2022-10-15

**Authors:** Mi Yao, Gang Yuan, Kai Lin, Lijuan Liu, Hao Tang, Jieying Xie, Xinxin Ji, Rongxin Wang, Binkai Li, Jiajia Hao, Huichang Qiu, Dongying Zhang, Hai Li, Shamil Haroon, Dawn Jackson, Wei Chen, Kar Keung Cheng, Richard Lehman

**Affiliations:** 1 General Practice Department, Peking University First Hospital, Beijing, China; 2 Institute of Applied Health Research, University of Birmingham, Birmingham, UK; 3 Department of Geriatrics, The First Affiliated Hospital of Sun Yat-Sen University, Guangzhou, China; 4 Family Medicine Center, The First Affiliated Hospital of Shantou University Medical College, Shantou, China; 5 Department of Cardiovascular, The First Affiliated Hospital of Sun Yat-Sen University, Guangzhou, China; 6 Department of General Practice, The First Affiliated Hospital of Sun Yat-Sen University, Guangzhou, China; 7 Nanfang Hospital of Southern Medical University, Guangzhou, China; 8 Shantou University Medical College, Shantou, China; 9 Shayuan Community Health Service Centre, Guangzhou, China; 10 Department of General Practice, Guangzhou First People’s Hospital, Guangzhou, China; 11 National Clinical Research Center for Respiratory Disease, The First Affiliated Hospital of Guangzhou Medical University, Guangzhou, China; 12 Faculty of Medicine, Macau University of Science and Technology, Macau, China; 13 Department of Endocrinology, The First Affiliated Hospital of Sun Yat-Sen University, Guangzhou, China; 14 College of Medical and Dental Sciences, University of Birmingham, Birmingham, UK; 15 Department of Nephrology, The First Affiliated Hospital of Sun Yat-Sen University, Guangzhou, China

**Keywords:** Communication, Training, General practitioners, Diabetes care

## Abstract

**Background:**

In China diabetes care is gradually shifting from secondary to primary care with great infrastructure investment and GP training. However, most GPs in China lack communication skills training, which is a huge obstacle in communication with their patients in primary care. In this study we seek to identify training priorities that is evidence-based, appropriate for the context of primary care in China, and that meet the real needs of both GPs and people with diabetes.

**Methods:**

A mixed method approach was used. A conceptual framework was designed based on the MRC framework, action research and adult learning theories. Through a systematic review of the literature and qualitative research with GPs and patients with diabetes, a list of communication skills training components was developed by the research team. A modified nominal group technique (NGT) with GPs was used to evaluate these contents. Purposive sampling was used to recruit a variation of participants (age, work area, practice years and education background) from general practices in Guangzhou city, China. Eight structured nominal groups were facilitated to elicit the views of group members, and participants rated the 9-point Likert scale of importance and feasibility of the training items independently, before and after focus groups. The ranking of each item was calculated, based on the mean Likert score ratings from all participants. Video recordings of four NGT group discussions were thematically analysed using the Framework Method to explore reasons for any differences in rating items.

**Results:**

29 males and 29 female GPs from 28 general practices participated in NGT group discussions, with a mean age of 38.5 years and mean 12.3 years of practice experience. Based on the mean scores of importance and feasibility rating scores, the top 3 ranked priorities for communication training were ‘health education’ (importance 8.39, feasibility 7.67), ‘discussing and explaining blood glucose monitoring’ (8.31, 7.46), and ‘diabetes complications and cardiovascular disease risk communication’ (8.36, 7.12). Five main themes were identified from focus group discussions through qualitative analysis: ‘impact on diabetes patients’, ‘GP attitudes towards communication skills’, ‘patient-related factors influencing the application of communication skills by GPs, ‘local contextual factors’, and ‘training implementation’.

**Conclusions:**

Priorities for communication skills training for Chinese GPs in diabetes care were identified. These are set in the context of GPs’ current experience of communication with patients in China who have diabetes, which is often unsatisfactory. This study describes the baseline from which better primary care for diabetes in China needs to be developed. Based on suggestions from GPs themselves, it identifies an agenda for improvement in communication as a key component of diabetes care in China.

**Supplementary Information:**

The online version contains supplementary material available at 10.1186/s12875-022-01868-8.

## Background

Diabetes is a common and costly long-term condition globally. It is estimated that 463 million people were living with diabetes in 2019 and this number is expected to increase to 578 million (10.2%) in 2030 [1]. Uncontrolled diabetes can lead to microvascular and macrovascular complications, disability, premature death and impaired quality of life [2, 3]. Diabetes affects more than 140 million Chinese people and is the sixth leading cause of death in China [4, 5]. However, diagnosis, treatment, and control of diabetes in China are not optimal [6, 7]. Adequate diabetes care is an urgent need in China to reduce the burden of diabetes and improve the quality of diabetes management.

Optimal diabetes care requires effective communication between health professionals and patients to achieve shared understanding of chronic illness and its management [8, 9]. Healthcare professionals should be competent in communication skills relevant to chronic disease management, and training is necessary to improve their skills [10]. Communication skills include active listening, showing empathy, shared decision making, and motivational interviewing, in order to understand patients, provide treatment opinions and facilitate the doctor-patient relationship and achieve better health outcomes [11].

There are three main diabetes care models in China including hospital-based care, community-based care, and a combination of both [12]. The hospital-based care model is very common and dominant in diabetes care. Diabetologists based in tertiary and secondary care hospitals are the main providers of diabetes care, with other specialists in cardiology, neurology, ophthalmology, and nephrology taking part in a multidisciplinary team. The care is hospital centred, mainly focussing on diagnosis and treatment in outpatients and inpatients [13]. Community-based care is based on general practitioners in primary care and promoted by the national chronic disease management plan by the Chinese government in recent years. A lot of community health service centres were built in cities and rural areas in recent years. There is a state service (contract) agreement between primary care teams and diabetes patients [14]. Community-based care mainly includes treatment, screening for diabetes complications, health education, establishing patients’ health records and supporting self-management. In the hospital-community combination model, hospitals have overall responsibility for the diagnosis and treatment of diabetes and screening and treatment of complications. Hospitals also have the responsibility for training GPs to improve their diabetes knowledge and clinical skills. Community health care providers are responsible for screening and following up high-risk patients with diabetes in their community. A referral system for diabetes patients is established between hospital and community services [15].

Although there are several diabetes care models in China, primary care based in the community has become a central point for diabetes management. This is because the rising burden of diabetes, diabetes complications, multimorbidity and the increasingly ageing population, has brought great challenges to a healthcare system with an over-reliance on secondary care. From the experience of several countries around the world, a well-developed primary healthcare system, where the majority of people with type 2 diabetes are managed, appears to be a good foundation for better clinical outcomes [16, 17]. This enables diabetes patients to receive timely, local access to medical support and holistic care.

Diabetes care is gradually shifting from secondary to primary care in China with great infrastructure investment and GP training over the past decades and in future as well. There is an implementation of health care reforms aimed at strengthening China’s primary health care system [18]. 400,000 new GPs will be trained by 2030, to produce a total workforce of 700,000, equivalent to 2–3 per 1,000 population [19]. However, GPs rarely receive communication skills training, which may impede effective communication with diabetes patients in primary care [20]. Communication skills training is needed that is evidence-based, appropriate for the context of primary care in China, and that meets the real needs of both GPs and people with diabetes.

To our knowledge, there are currently no effective training programs on communications skills for GPs in diabetes care in China. Educating Chinese GPs in this area may represent a colossal task, and our early qualitative research with GPs and patients suggested many different areas for development in communication [21, 22]. Attempts to cover this breadth within a training curriculum risks a superficial approach to a complex phenomenon, where knowing ‘where to start’ may be difficult. Our systematic review suggested that such that ‘one size fits all’ approaches to communication skills training should be exercised with caution [23]. Furthermore, the training of communication skills for Chinese GPs may represent a paradigm shift in learning and patient care, which could be overwhelming for many learners, risking disinterest or disengagement. This research focuses on identifying important ‘next steps’ for GP education in China on communication skills for patients with diabetes; aiming to foster engagement amongst GPs amidst significant resource constraints.

## Methods

### Study design

**Fig. 1:**
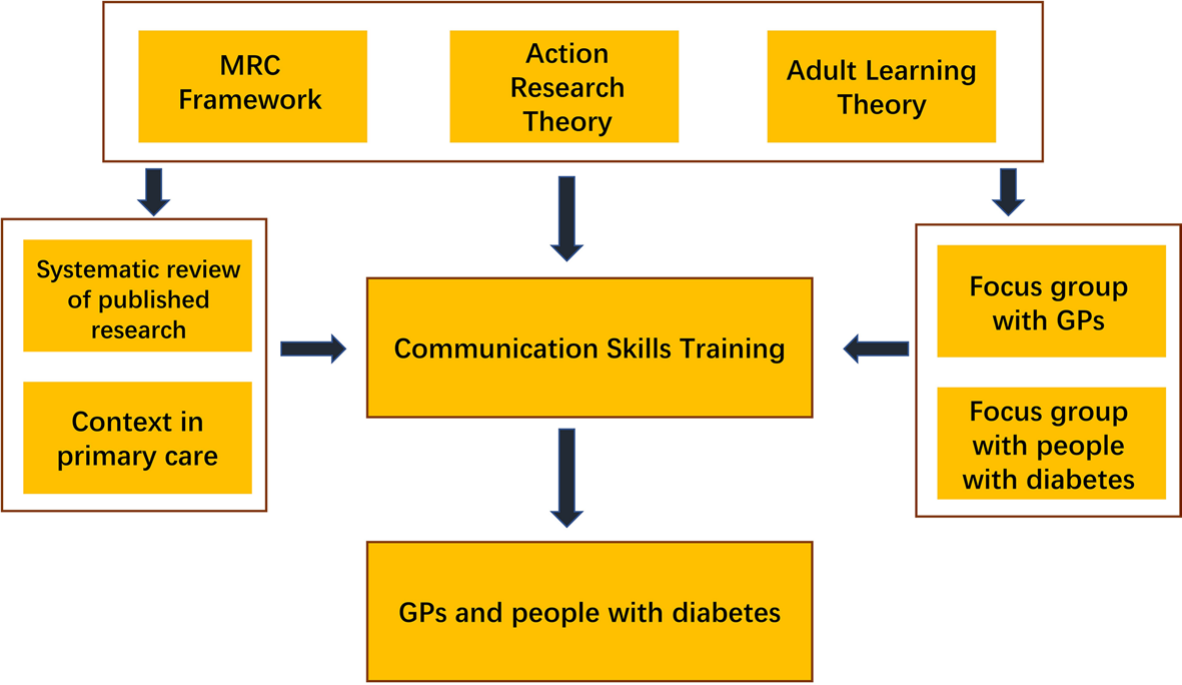
Conceptual framework of this study

As communication skills training is a complex intervention with multiple components, the MRC conceptual framework was considered to encourage good quality [24]. Developing training programs for GPs is an educational activity that involves interaction between designers, educators, and learners. For researcher involvement in the process, we used the methods of action research [25]. GPs had their medicine degrees from universities or colleges, qualifications in general practice and clinical practice experience in their workplace. We applied adult learning theories to understand their learning and practice behaviours, especially in a changing and reforming primary health care system context [26]. With learning from those theories, a theoretical framework was developed to guide this mixed method research by a systematic and iterative approach to identify and refine communication skills training programs for GPs in managing diabetes patients (Fig. 1).

As outlined in the background section above, a mixed method programme of research was undertaken iteratively, with each stage of research building on the previous. A systematic review was conducted on randomized controlled trials on the effectiveness of communication skills training for healthcare professionals on the outcomes and experience of patients with diabetes worldwide. Key elements for successful communication training were identified including teaching theories, appropriate training content, and training methods. Research gaps in communication skills training for diabetes care were identified in this systematic review: training needs to understand what health professionals and patients’ thoughts and goals should be based on encouraging patients to self-manage and shared understanding of diabetes management [23].

Two focus group studies were then conducted to explore and provide deeper insights into the problems of communication between GPs and people with diabetes and relevant training issues for GPs. 4 focus groups with 15 GPs and 5 groups with 22 diabetes patients were involved. Insights of their experiences, perceptions, behaviour, and views on the barriers and facilitators to delivering diabetes care were gathered from two focus group studies from a wider primary health care system perspective. Rich themes emerged from both GPs and patients’ perspectives. GPs described several difficulties in communication with their diverse patients. Patients expressed that they needed more information and better communication channels with their GPs. Participants acknowledged that aspects of the health system were obstacles to good communication, such as insufficient consultation time and a consultation environment.

**Fig. 2:**
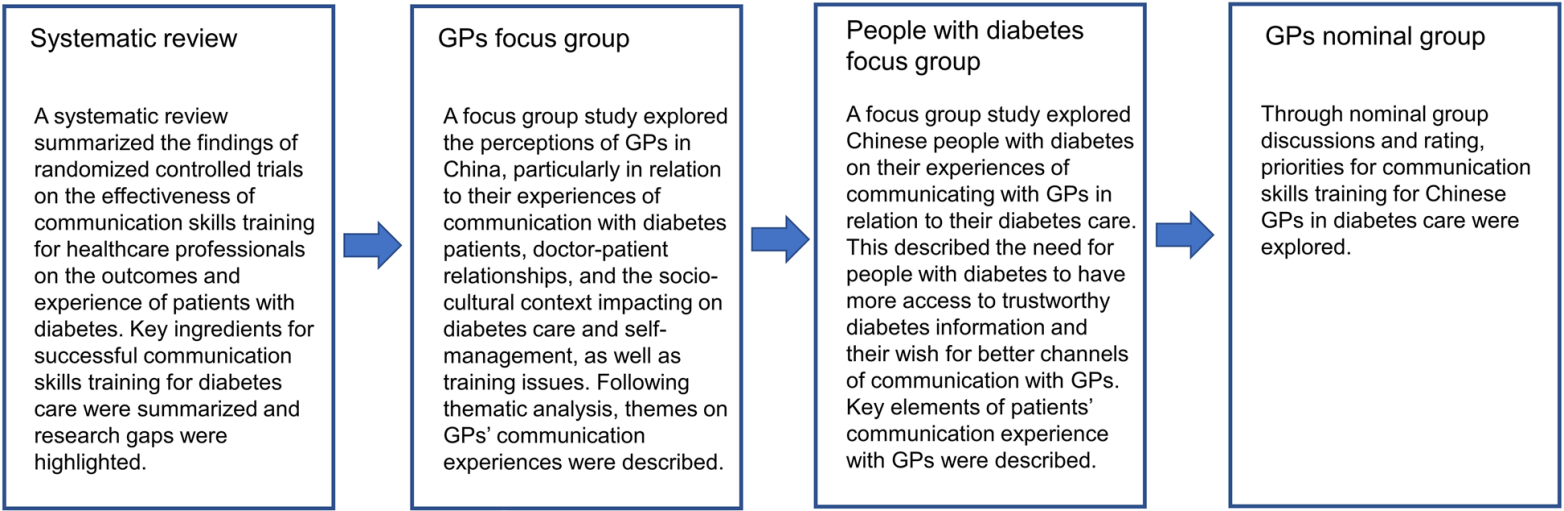
Flow diagram of different stages of the study

Data from the above studies were combined to inform potential communication skills components for training. In this article, we specifically reported the details of using nominal group technique (NGT) to evaluate, refine and rate these components. The NGT is a method of eliciting and aggregating judgments in a transparent and structured way. It can provide important information on levels of agreement between participants [27]. At the outset of designing a training programme for a complex phenomenon (which may be largely unfamiliar to learners, or where there is a suggestion of learner disengagement), this method offered a means to begin to identify areas of priority for those who will subsequently embed these skills in their practice. The NGT is widely used in health care service research and health education [28]. It can also provide a range of opportunities to better understand the reasons for the opinions and judgements of others, providing scope for the identification of new or unconsidered themes [29].The different stages of the study are shown in Fig. 2. Good Reporting of A Mixed Methods Study (GRAMMS) was used to report this study [30].

### Identifying a list of potential communication skills training components

A list of communication skills training components was developed for discussion and review before the NGT focus group. Based on the systematic review of the literature, and qualitative research with patients and GPs, a provisional list of communication skills training priorities was developed by researchers (MY & GY). The systematic review identified several communication skills training contents used from previous trials and showed impact on diabetes care [23]. The qualitative studies illuminated the importance of context in implementing communications skills training, especially the socioeconomic and health care system background in China [21, 22]. These items, along with their descriptions, are outlined below in Table 1**.** We critically reviewed and analysed the evidence from the academic literature and provided refinement on terminology and descriptions for each of the identified training components. As outlined previously, our previous research with GPs and patients had highlighted both ‘importance’ and ‘feasibility’ as key areas to consider, and these were chosen as particular areas of focus within the Nominal Group ranking approach.

**Table 1:** Potential components for communication skills improvement

Item	Potential components for communication skills improvement (for training)	Sources of evidence		Description
		**Findings from systematic review**	**Findings from qualitative studies**	
1	Active listening	√	√	Listen attentively to the patient’s opening statement, without interrupting or directing the patient’s account. When asking questions, leave space for patient to think before answering, or to pause for thought before going on
2	Express empathy	√	√	Deliberately show your understanding and appreciation of the patient’s feelings or predicament; overtly acknowledge patient’s views and feelings
3	Share bad news		√	Become skilled at breaking bad news to patients who have started or already developed complications, such as a diagnosis of diabetic nephropathy, retinopathy, or associated foot problems. Giving bad news is a complex challenge in communication that involves a series of preparations and steps
4	Use examples		√	Use examples to share relevant information with diabetes patients and help their understanding by using materials such as stories or pictures (such as pictures of diabetic foot problems)
5	Idea, concerns and expectations		√	In people with diabetes, explore their beliefs, their concerns about current problems and how these problems affect them. Ask about their expectations for solutions, and their willingness to take personal action to achieve them
6	Nonverbal skills: body language, facial expressions, eye contact, speed, tone, and silence		√	Convey and receive information and understanding in ways outside direct verbal communication
7	Negotiation of behavioral change	√	√	Use negotiation as a method to help patients make lifestyle changes (such as addressing obesity, adherence to treatment, smoking cessation, and physical activity) to improve their health
8	Evaluate the patients’ confidence, support patients’ self-efficacy and optimism	√		Assess the individual's confidence in his or her own ability to perform specific tasks required to reach a desired goal. To cope effectively with the complex demands of the diabetes treatment regimen, a sufficient sense of self-efficacy is required. Self-efficacy is a dynamic, changeable belief, which may be enhanced by behavioral interventions, resulting in an increased motivation for behavioral efforts
9	Motivational interviewing	√	√	Use motivational interviewing (MI) as a person-centered strategy to guide patients towards changing a specific negative behavior. There are four processes: 1) engaging, which requires an understanding of the patient's point of view to develop a working alliance with them; 2) focusing, the process of developing one or more clear goals for change; 3) evoking, calling forth the patient’s own motivation for, and ideas about, change; 4) planning, which involves the collaborative development of the next steps that the individual is willing to take
10	Shared decision making	√		Shared decision making is a key component of patient centered health care. It is a process in which clinicians and patients work together to make decisions and select tests, treatments and care plans based on clinical evidence that balances risks and expected outcomes with patient preferences and values. There are four major processes: 1) clinician informs patient that decision is to be made and patient’s opinion is important; 2) clinician explains the options and the pros and cons of each (relevant) option; 3) clinician and patient discuss patient preferences and clinician supports deliberation; 4) clinician and patient discuss the patient’s wish to make the decision and discuss follow-up
11	Discuss blood glucose monitoring and explanation		√	Carefully communicate blood sugar figures with patients, and guide patients to consider the significance of different indicators based on evidence. Be aware of tension, anxiety, depression, and other emotions caused by fluctuations in blood sugar or glycosylated hemoglobin and seek to reduce these
12	Diabetes complications and cardiovascular disease risk communication	√	√	Discuss the risk of complications such as problems with the heart, kidneys, and eyes and how these can be reduced by an adequate treatment with medication and by adopting a healthy lifestyle. Learn how to help patients understand the risks of developing severe diabetes related complications to enable them to make informed choices. It is important to provide a clear and very simple message, tailoring the explanation of risk and frequency statistics in a way that the patient can understand, such as using visual aids or discussion of absolute risk across a 10-year period. Messages about risk should consist of information on what causes the risk, the consequences of the risk, and what can be done to prevent or treat the problem. Positive framing, by highlighting the benefits of behaviour change (rather than focusing on the effects of not changing), appears more likely to increase patients' motivation
13	Medication adherences	√	√	Look out for poor medication adherence, by checking on whether prescriptions have been requested and dispensed, and by asking patients directly. Poor adherence can be linked to key nonpatient factors (e.g., lack of integrated care in many health care systems and clinical inertia among health care professionals), patient demographic factors (e.g., young age, low education level, and low-income level), critical patient beliefs about their medications (e.g., perceived treatment inefficacy), and perceived patient burden regarding obtaining and taking their medications (e.g., treatment complexity, out-of-pocket costs, and hypoglycemia). There are several communication skills: 1) elicit patients` beliefs (e.g. perceived benefits and harms of taking medicines); 2) assess patients’ medication adherence; 3) assist patients’ in overcoming barriers to treatment adherence (include discussing healthcare system issues); 4) ask patients to generate and write down the exact circumstances in which they would take their medication. Be aware that poor adherence to treatment may be a signal for other psychosocial problems (see Sect. 16)
14	Follow up or referring	√	√	Know when to refer diabetes patients to endocrinologists and how to make appropriate communication, in line with local guidelines and in accordance with patient wishes. Ensure that you coordinate different doctors' diabetes treatment plans and arrange regular follow-up of diabetes patients with specific time
15	Cultural biases and patients background awareness	√	√	Be aware that patients from different regions (such as urban and rural areas) may have different perceptions of diabetes and treatment options, and it is necessary to consider the patient's background, family or economic factors and other problems that bring difficulties to diabetes patients. The dialect used by patients is also a cultural difference, and some patients prefer their doctors to communicate in dialect
16	Explore the patient's emotional and psychosocial (mental health) problems		√	Specifically ask about psychosocial problems in diabetes patients, which often result in serious negative impact on patient's well-being and social life, if left un-addressed. Patients can feel overwhelmed with the demands of self-management. Feelings of frustration, fatigue, anger, burn out, and low mood can be experienced due to complexities in the routine of self-management of the control of blood sugar. Family members may not understand the feelings of the patient, and food differentiation and restriction of food by family members may lead to further distress. Avoid the over-simplification of a label of ‘noncompliance to treatment’. It is important to incorporate psychological screening and management at every level of diabetes care
17	Use online or telephone communication technic		√	Make use of online communication, or text communication, in line with what suits each patient best in each situation. Online communication is becoming more and more common, making it easier and faster for patients to find and call doctors, reduces unnecessary travel time, and costs, and also increases the frequency of contact with doctors. Online communication, or texting communication, is very different from face-to-face communication, particularly as non-verbal communication between doctor and patient can be restricted. When interacting online, active listening, multiple acknowledgements, and positive responses are essential for online communication
18	Health education		√	Develop skill in sharing diabetes-related health knowledge with patients in various forms, e.g., written material, online resources etc. Be aware of different knowledge sources and ensure that those used by your patients are reliable, safe, and up to date. When discussing topics, check on your patient’s knowledge and sources of advice
19	Patient held health record management		√	Each time the patient visits, primary care physicians acquire the patient's personal health record book, consult the previous medical information, and record the information of this visit, so that the patient can use one patient's personal health record book to record the condition of diabetes in different hospitals as far as possible

However, through the creation of conditions for participants to discuss and reflect upon their ranking of training components, we also aimed to leave sufficient flexibility for new themes, ideas and priorities to develop. This was captured through facilitated group discussion.

### NGT Participant recruitment

We recruited GPs from a Guangzhou GP training program which began 2019 and is supported by the Guangzhou Municipal Health Commission [31]. This training program mainly focuses on the improvement of GPs’ clinical skills, with the help of specialists from hospitals in Guangzhou. Purposeful sampling to obtain variation of demographic (age, sex, practice location, practice years and education background) was used to select 60 GPs in this program from 30 community health service centers in 11 districts (both urban and suburban) throughout Guangzhou city [31]. Guangzhou is a modern industrial city located in the South of China. It is the capital city of Guangdong province with close to fifteen million urban residents at the end of 2019 [32]. There were 188 community healthcare service centers (general practices) with about 5000 GPs at the time of the study [33]. All GPs were qualified in general practice and had more than 3 years of work experience as required by the program [31]. On average, participants in this program received one full day of training per month.

We aimed to invite all 60 GPs to take part in our 2-h NGT focus group. We telephoned and emailed them with information about the study and the NGT process with the support of Guangzhou GP training program organizers. 58 GPs agreed to take part in the NGT group and provided electronic informed consent. Eight parallel groups were hosted, with the group size ranging from six to eight members. There was no compensation offered to participants. Ethical approval was given by the Medical Ethics Committee of the First Affiliated Hospital of Sun Yat-sen University (Reference number [2019] 369).

### NGT focus group and data collection

Eight facilitators (MY, LL, KL, BL, GY, HT, RW, JX and LB) in research team, two for each NGT group, were trained in NGT and were familiar with all the study aims and methods. A facilitator protocol was developed. A pilot NGT group with facilitators was conducted and minor adjustments were also made to the descriptions of training components based on their feedback.

Eight structured focus groups were subsequently held virtually with GP participants between January and February 2021. GPs working in similar geographical locations were assigned to the same team, with 7 or 8 GPs in each team. Each NGT group met virtually via a web videoconferencing platform (Classin, https://www.classin.com/en/) which participants were proficient in.

Prior to each focus group, we sent out an information pack describing the NGT process. Participants were asked to independently review the list of communications skills training components one week prior to the online NGT focus group, rating each component on 9-point Likert scales. Two questions were posed: 1) importance: how important would this communication skill be to train GPs in? (Rating of 1–9, not at all important = 1; very important = 9); 2) feasibility: how difficult or easy would this strategy be to implement into GP training? (Rating of 1–9, very difficult = 1; very easy = 9). Every participant submitted their ratings by email before attending focus groups. Participants were also invited to share free text comments and were required to complete an anonymized questionnaire capturing demographic characteristics.

**Fig. 3:**
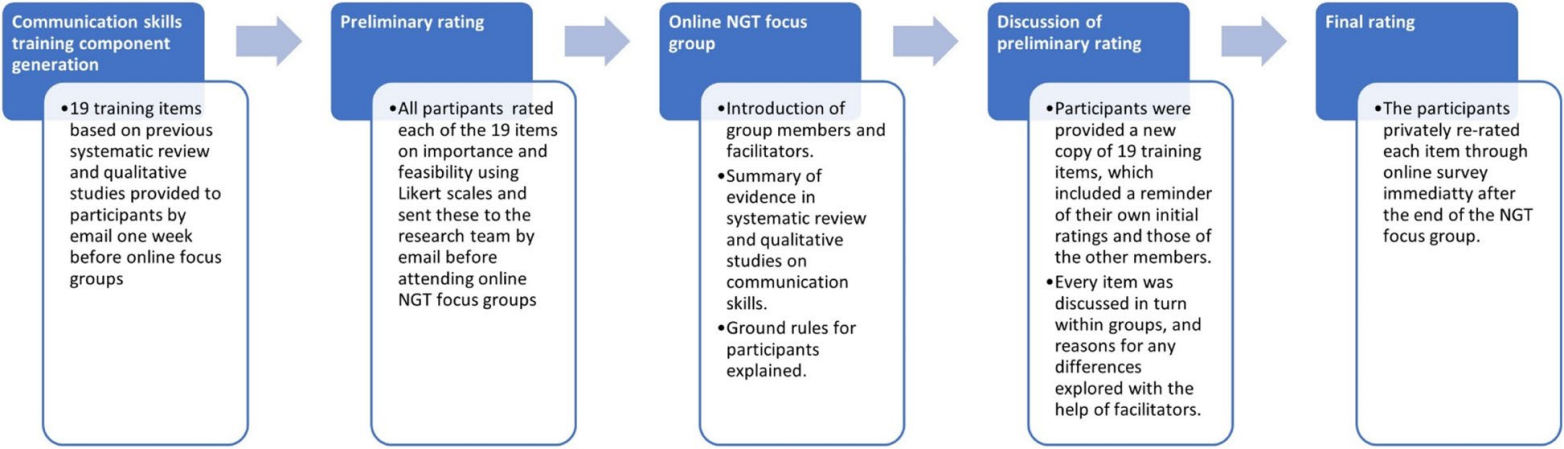
Nominal group technique process for the study

The structure for each group discussion is outlined in Fig. 3, and each group took 2 h. At the beginning of the focus group session, facilitators provided a brief summary of our systematic review and qualitative studies. The facilitator briefly described the listed communication skills training components, giving each component equal time and emphasis to avoiding favoring towards any particular one. The facilitator aimed to encourage discussion among quiet members of the group and made sure the group discussion was focused on evaluating each of the training components. All facilitators were trained in nominal group technique and had no prior relationship with any of the participants.

At the online focus group, preliminary voting results were presented on a Microsoft Excel spreadsheet, which outlined a reminder for each participant of their own initial ratings, and also contained those of the other members. Every item (rated for both importance and feasibility) was discussed in turn, and reasons for any differences explored. Participants were asked two questions to explore differences in the rating of items: 1. What do you think of this item? 2. Please take a look at the scores given by others in your group and your own scores (importance and feasibility). Is there a big difference and if so, why?

The participants then independently re-rated each item immediately at the end of the group discussion, using an online survey. The qualitative data of group discussions was collected by video recording of the focus groups and field notes were made by facilitators.

### Data analysis

All quantitative data were analyzed using STATA 16, including participants’ demographic characteristics and the Likert ratings of importance and feasibility of the training components. The mean score of the feasibility and importance ratings for each item were presented in bar charts. A ranking of each item was subsequently calculatedbased on the sum of mean scores in feasibility and importance.

For qualitative data, two of the researchers (MY and XJ) initially reviewed the entire transcripts of the 8 NGT group discussions. Transcripts were imported into NVivo12 software and coded independently by two researchers (MY and GY). Data were analyzed inductively by thematic analysis based on the Framework Method. Researchers independently read transcripts and open-coded the data by marking and categorizing key words and phrases to generate the initial codes. Meaning units from the transcripts were discussed and condensed to a description close to the context. Discrepancies and disagreements were resolved through discussion and consensus to develop the initial thematic framework, which was then applied to the remaining transcripts. This process was continued until no new codes emerged (data saturation), which happened after the analysis of four transcripts. Similar codes were grouped to form broader themes by constant comparison until themes and subthemes were developed. The themes that emerged from analysis of the first four transcripts analysis were checked against the transcript of a remaining, randomly selected group. The themes were presented to other team members for further discussion to reach a consensus.

## Results

Eight NGT groups with 58 GPs from 28 general practices in Guangzhou (mean duration 95 min, range 85 to 100) were held and no participants dropped out. See Table 2 for GP characteristics. Details of NGT group information were shown in supplementary table.

**Table 2:** Characteristic of participants (*n* = 58)

Characteristic	No. (%)
Sex	
Male	29 (50%)
Female	29 (50%)
Age	
30–40 y	37 (64%)
41–50 y	20 (34%)
> 50 y	1 (2%)
Practice location	
City center	37 (64%)
Rural or suburb	21 (36%)
Practice years	
< 10 y	21 (36%)
11–20 y	28 (48%)
> 20 y	9 (14%)
Education background	
College degree	2 (3%)
Bachelor’s degree	49 (84%)
Master’s degree	7 (13%)
Professional title	
Physician	4 (7%)
Attending physician	34 (58%)
Associate chief physician	17 (30%)
Chief physician	3 (5)

### Nominal group ranking

**Fig. 4:**
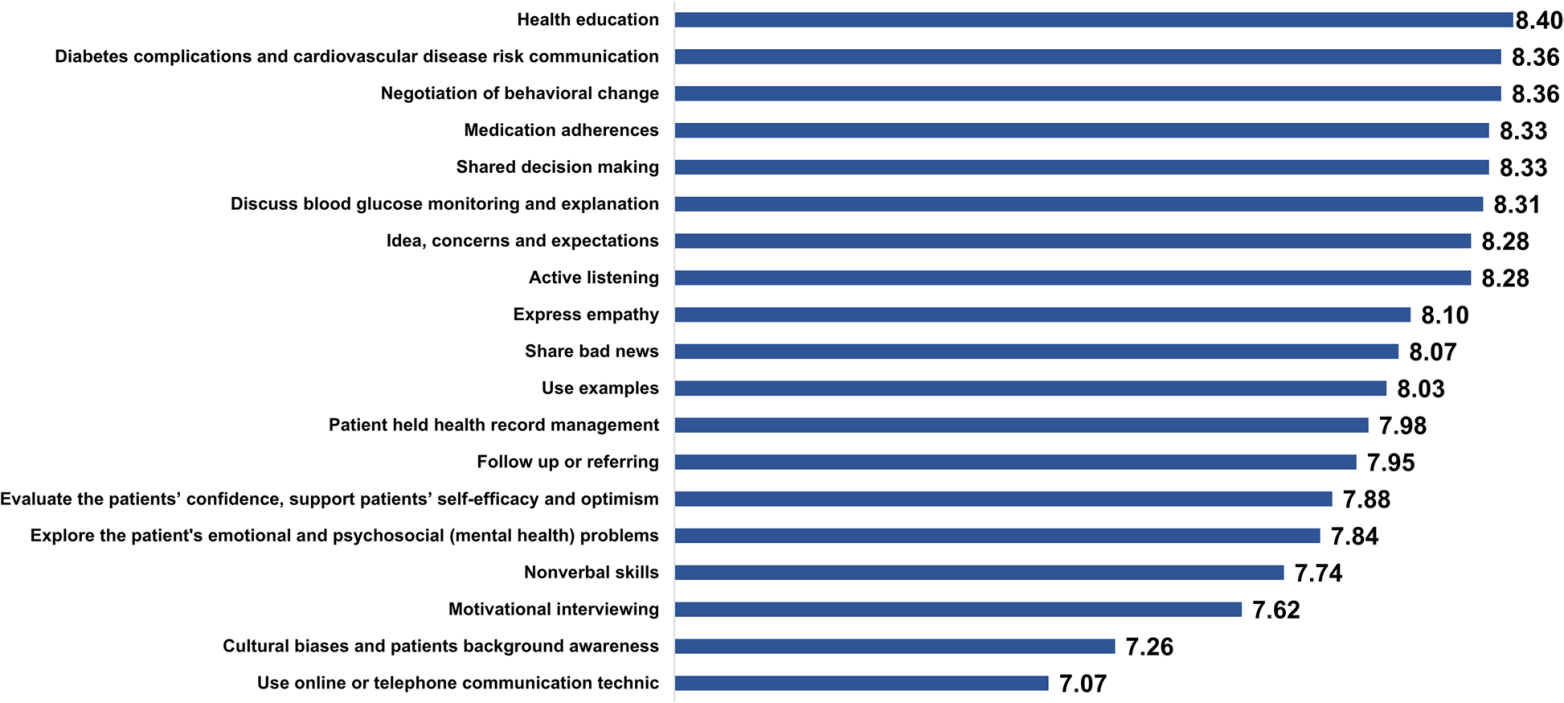
Mean scores of importance for each item

**Fig. 5:**
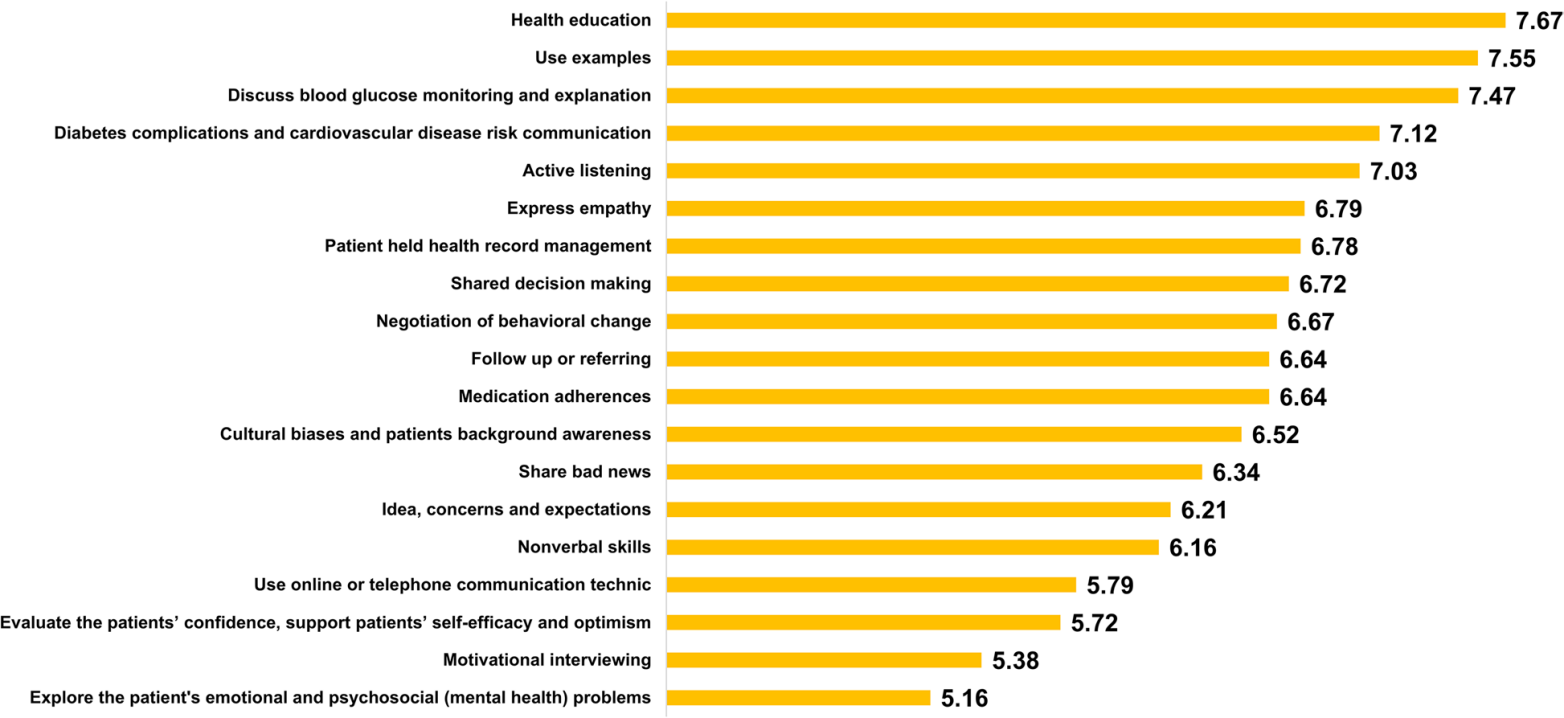
Mean scores of feasibility for each item

Figures 4 and 5 outlines the importance and feasibility mean scores for the various training components. In terms of importance ratings, the top 10 training components, based on importance ratings were ‘health education’(8.40), ‘diabetes complications and CVD risk communication’(8.36), ‘negotiation of behavioral change’(8.36), ‘shared decision making’(8.33), ‘medication adherence’ (8.33), ‘discussing and explaining blood glucose monitoring’ (8.31), ‘active listening’ (8.28), ‘idea, concerns and expectations’ (8.28), ‘expressing empathy’ (8.10) and ‘sharing bad news’ (8.07). Based on feasibility ratings, the top 10 were ‘health education’ (7.67), ‘using examples’ (7.55), ‘discussing and explaining blood glucose monitoring’ (7.47), ‘diabetes complications and CVD risk communication’ (7.12) ‘active listening’ (7.03), ‘expressing empathy’ (6.79), ‘patient held health record management’ (6.78), ‘shared decision making’ (6.72), ‘negotiation of behavioral change’ (6.67), ‘medication adherence’ (6.64), and ‘follow up or referring’ (6.64). The top 3, based on the sum of mean score of importance and feasibility, were ‘health education’ (importance 8.39, feasibility 7.67), ‘discussing and explaining blood glucose monitoring’ (8.31, 7.46), and ‘diabetes complications and CVD risk communication’ (8.36, 7.12). Details of score and ranking for each component in the NGT groups were shown in supplementary table.

### Themes

Five main themes emerged out of the group discussions: impact on diabetes patients, GP attitudes towards communication skills, patient-related factors influencing GP communication skills, local contextual factors influencing GP communication skills, and factors related to communication skills training program implementation. Illustrative quotes are presented for each theme in Table 3 and help to explain the quantitative ranking results.

**Table 3:** Select GPs Quotations for each theme explaining the reasons for the ranking results

Subthemes	Quotations
	**Impact on diabetes patients**
Patients understanding of condition	“It is best for patients to understand their condition, such as the severity. When they do not understand, we will give a simple example, so that they can understand the disease, the treatment and progress. They also can better cooperate with our treatment.” (GP 12, Group 2, item 4)
Long-term cooperation with doctors	“There was a patient who came in with breast cancer and diabetes. She was very secretive. She did not want to people know she had breast cancer. But when I started talking to her, she told me that she did. And then her tears came out. She said no one cared about her. She had seen diabetes for so many years that no one cared about her comorbidities and complications. Then I saw how sad she was, and I held her hand. And then there was a silence, she said a lot of her worries. I just listened and did not give a lot of guidance, because after all, I was not very good at breast cancer treatment. From then on, this patient only came to see me once a month. She did not go to clinics when I'm resting or when I’m out of the clinic. Therefore, I think this skill is very important, because the patient will understand your caring, patient will be in close contact with you, and will be more compliant to your opinions.” (GP 20, Group 3, item 4)
Patients' experience improvement	“I tell my patients a lot of things to encourage them. Life is a state of mind. Even the same disease, same symptoms, maybe this person thinks it's okay and he's going to have a very fulfilling life. But for some people, it is like the sky is falling in. So, I think communication is very important, it can explore patient's attitude towards life, as well as improve his experience with diabetes. I want my patients to be optimistic. No matter what kind of diseases or difficulties they face, I will teach them such a positive thought by using communication skills.” (GP 11, Group 2, item 8)
	**GPs attitudes towards communication skills**
Seldom using communication skills	"In practice, we really ignored them. We did not do enough." (GP 1, Group 1, item 5 and 16)
Essential competencies	“Active listening, expressing empathy, sharing bad news, using examples, are skills that went on almost every day in our daily work, and I think it should be basic competencies for every doctor.” (GP 20, Group 3, item 1, 2, 3 and 4)
Mutual understanding	"If good communication skills used during consultation, it will be easy to build a common understanding with diabetes patients. They can feel that you are caring. " (GP 18, Group 3, item 8)
	**Patients’ factors influence on application of communication skills**
Personality	“Different levels of patients have different ideas, concerns, and expectations. We need to observe and understand the patient's background to know how to communicate with them.” (GP 26, Group 4, item 5)
Health literacy	“Many patients have different levels of awareness of diabetes, especially in the urban and rural areas, and most of them are not well educated. Sometimes, when explaining his condition to him, such as medication, the patient thought that his blood sugar was well controlled, he would stop the medication on his own, and would not follow the doctor's advice. It will take a long time for doctors to work in and communicate with him before things get better.” (GP 5, Group 1, item 13)
Aging population	“Most of the patients I care for are the elderly, and their desire to talk is very strong. Even some old people come to me, they neither want to prescribe medicine or cope with symptoms. They just want to talk to me. So, I think it's important to listen to patients.” (GP 28, Group 4, item 1)
	**Local context factors influence on application of communication skills**
Insufficient time	“I feel it is quite difficult. In our general practice, one doctor sees dozens of patients in the morning. And if each patient wants to say everything, there is definitely not enough time.” (GP 16, Group 2, item 9)
Regional differences	“The electronic medical record system is far from perfect. Only in our own clinic patients’ records can be traced. But here we have a higher population floating (migrant population), for example, patients who do not always live in this area, they may have gone to another village or community. It would take a long time to retrieve the patient's records from other medical institutions. Sometimes even more than half an hour spent, there is no guarantee of a result. Even if we could retrieve the patient's records, things in our hands were not what we doctors wanted.” (GP 16, Group 2, item 19)
Healthcare resource, policy and guidelines	“Even if we are trained to recognize anxiety, depression, and other mental health issues, we don't have the capacity to help them. At best, we just comfort him with words, right? To talk to him about life matters, only to this level. When it comes to medication, there are not enough medicines in our community health care service. Doctors have no experience in using drugs and are afraid to give them to patients. If I find that the patient has mental problems that need to be referred, I find that I don't know how to answer this question, and I don't have a good way to help him. That is to say, how do I help patients to refer patients to which hospital, which department, which doctor? Basically, there is no system of referral.” (GP 27, Group 4, item 14, 15)
	**Factors involved in communication skills training program implementation**
Previous training experience	“It is difficult to master this skill aimed at improving patient adherence, and there is no previous training in this aspect.” (GP 5, Group 1, item 9,13)
Trainees' gender difference	“In my opinion, it may be better for female doctors to show empathy. Sometimes, male doctors may not easily show their feelings or emotions as well as speak out. Female GPs trained have advantages in using those skills.” (GP 4, Group 1, item 2,6)

### Impact on diabetes patients

Most GPs tended to believe that using a variety of communication skills in medical encounters can promote better understanding of diabetes among patients as well as long-term cooperation in treatment. Participants thought that communication skills had an impact on the healthcare experience of diabetes patients and their confidence in self-management, as well as better addressing their information and psychological needs. They felt that these needs, in turn, impacted patients’ choices about their lifestyle and their family’s ability to support the management of their condition. Enhancing the healthcare provider-patient relationship and trust were also mentioned by most GPs.

### GPs attitudes towards communication skills

Most GPs reported that they tended to ignore the importance of, and were reluctant to focus on, the use of communication skills in their day-to-day clinical practice. They generally lacked training opportunities to learn and acquire knowledge on communication skills. They were also unaware of a number of communication skills relevant to chronic disease management, such as motivational interviewing and shared decision making. However, some believed that some communication skills were essential competencies and were integrated into their daily work. The patience of GPs, their experience, mood, interest, and clinical workload were all factors that were deemed to influence the use of communication skills.

Perspectives on the 19 items for communication skills training differed. Some skills were regarded as too complex to handle with, too many evaluation scales or tools to adopt, or too many steps to process, such as exploring diabetes patients’ emotional and psychosocial problems, evaluating patients’ confidence, and risk communication. However, other skills were believed to be easy to follow, such as discussing and explaining blood glucose monitoring. Many GPs mentioned that using communication skills, such as breaking bad news or risk communication with diabetes patients, was especially tricky to strike a balance between not panicking the patients and not making them overconfident. Some GPs emphasized that patients’ participation and mutual understanding were necessary.

### Patients’ factors influence on application of communication skills

Most GPs described several patient-related factors that impacted on the use of communication skills during clinical encounters. Participants noted differences among their patients, including age, personality, psychosocial and family background, health literacy and economic income. Some GPs believed that diabetes patients’ knowledge was often insufficient, with frequent misunderstanding of the condition and its management. Patients were perceived to be reluctant to express their feelings and inner thoughts regarding their condition. They were also reluctant to change their lifestyle behaviours (such as exercise and diet) and routine medication even if it was recommended by their healthcare professional.

Some participants believed that older patients had a strong desire to express their complaints, concerns, and expectations, but that this was often communicated in a disorganized and unfocused fashion. Participants felt that the discussion of blood glucose figures often opened up opportunities for discussion, as patients put great importance on whether their figures achieved goals or not. GPs believed that developing skills to support patients’ optimism and confidence were difficult.

### Local context factors influence on application of communication skills

Participants thought that the 19 items of communication skills applied to clinical encounters with diabetes patients were hard to operationalize due to local context. Most GPs mentioned that they had insufficient time to engage in communication with patients. In their practices, it was always crowded with patients in almost all service delivery points. Administrative workload (e.g., electronic healthcare records) occupied large amount of doctors’ time. GPs discussed that it was hardly realistic to adopt time consuming or implementing multistep communication skills, especially motivational interviewing, shared decision making and exploring patients’ emotional problems. Regional differences (e.g., urban and rural areas, migrant population), local policies (e.g., guidance or clinical guidelines), healthcare resource (e.g., access to medication and information system) and coordination with hospitals were also factors influencing communication skills using.

### Factors involved in communication skills training program implementation

Many GPs described that several factors influenced training programs communication skills improvement. Almost no participants had previous training experience on certain communication skills, such as motivational interviewing, shared decision making, exploration of patients’ emotional problems, evaluation of patients’ confidence and support for optimism, although some GPs were interested in these areas. GPs felt that training periods should be for sufficient duration (over a longer term), incorporating continuous learning cycles, with opportunities to embed learning in practice. Various methods of training were proposed by GPs, including interactive teaching, role play and sharing clinical cases, and there was general consensus that a detailed training method could be appropriately found.

Participants felt that some skills, such as discussing blood glucose monitoring and explanation, diabetes complications and cardiovascular disease risk were flexible to learn, easy to embed and they felt able to acquire relevant skills. For nonverbal skills and expressing empathy, some male GPs felt that they might be less proficient than their female colleagues because they were less willing to acknowledge and express their own feelings and emotions.

## Discussion

Our study described the methods for adapting a priority technique using NGT combined with focus groups for development of a communication skills training program in diabetes care for GPs in China.

Through the early stages of our qualitative research with patients and GPs, it became apparent that GPs faced considerable time and resource constraints, which risked disengagement with implementing elements of quality communication in their interactions with patients. Building on these findings, the NGT arm of our study enabled us to identify areas of communication that were deemed both important and feasible to GPs. We have identified core themes of high priority to GPs in China. These include health education, discussing and explaining blood glucose monitoring and explanation, and diabetes complications and CVD risk communication.

Previous clinical trials in training physicians in communication skills to improve health care outcomes failed to show definite benefit [23], and we would suggest that the NGT approach offers a means to identify what might be improved if educators identified the specific needs and ideas from the trainees themselves.

Several reasons affected the prioritization from the qualitative analysis of NGT groups. Although most GPs tended to believe that using various a variety of communication skills in medical encounters can promote diabetes patients’ better understanding of diabetes and improve diabetes management, several factors arising from doctors themselves, their patients and the external environment impede their potential needs from being met in training. They acknowledged that different aspects of communications skills were too burdensome or complex to implement. For some skills, such as motivational interviewing and shared decision making, most of GPs had no conception and had not even heard of them until they participated in this study. They also had no previous training experience in terms of a lot of communication skills, which may have made them choose more familiar items when rating. The reasons why GPs prioritize the top 3 of communication skills as health education, discuss blood glucose monitoring and explanation, and diabetes complications and CVD risk communication might be that they were able to acquire relevant knowledge with relative ease. And when learning or using such skills, they had guidelines for reference which were relatively objective, clear, and explicit. But although GPs discussed that it was hardly realistic to adopt time consuming or multistep communication skills, such as exploring diabetes patients’ emotional and psychosocial problems, and evaluating patients’ confidence, those skills still had a relative high ratings and rankings.

Results from our study have some similarities with previous studies. Low health literacy is common among diabetes patients and has been associated with lacking diabetes knowledge and possible worse health outcomes [34]. Being capable of good health education skills in clinical encounters can be seen as a means for improving patients health literacy [35]. In our studies, we define the following core health education skills: sharing diabetes-related health knowledge with patients in various forms, e.g., written material, online resources etc.; being aware of different knowledge sources and ensure that those used by your patients are reliable, safe, and up to date; when discussing particular topics, checking on the patient’s knowledge and sources of advice.

Our findings also can be explained by the low health literacy of diabetes patients, as well as the low health education ability of general practitioners in China. Numeracy skills are also important for diabetes patients, which means the ability to understand numbers in the measurement, estimation, and risk [36]. Evidence from other studies suggests that there is an important association between patients’ numeracy skills and glycemic control [37]. There is no doubt that discussing blood glucose monitoring and explanation, and diabetes complications and CVD risk communication is closely related to patients’ understanding of numbers. It is important for GPs to provide a clear and very simple message, tailoring the explanation of risk and frequency statistics in a way that is suitable for current patients’ numeracy in China.

Our results suggest several considerations for educators tasked with the design and delivery of teaching programmes for communication skills. Before implementing a teaching programme, we must first understand the resources, constraints, and opportunities of the clinical context. Our results, which indicated regional and local variation in resources and practice, indicate that a teaching programme for communication skills in diabetes in China will need to incorporate sufficient flexibility for local modification. Although less familiar to medical learners in China, providing teaching formats that invite interaction, such as role-play, interactive learning or sharing cases were acceptable to our research participants and may offer sufficient scope for learners to consider how they would adapt their learning for their local context.

GPs in China seldom received communication skills training in their medical school or continuing medical education for residents. Even in the few training programs implemented in China, most use oral presentations as a strategy and with self-designed assessment tools by educators to evaluate the quality of programs [20]. More importantly, GPs do not receive feedback about their interactions with patients once they have left medical school or residents training. This is supported by the experiences and views expressed by GPs in our study. It is also possible that GPs lack incentives (e.g., a certification, rewards, or personal development) to participate in training programmes to develop communication skills from the perspective of a behaviourist learning theory or cognitive learning theory.

Focusing on elements that are deemed both important and feasible to learners may offer a useful starting springboard for teaching programme design. In our context, this relates ‘health education’ (importance 8.39, feasibility 7.67), ‘discussing and explaining blood glucose monitoring’ (8.31, 7.46), and ‘diabetes complications and CVD risk communication’. Whilst we recognise that these elements may vary across countries, conditions and health settings, we have found it useful to engage with learners using NGT to shape teaching content in an area of clinical practice that is largely unfamiliar to Chinese GPs. This has the potential to subsequently foster greater engagement with subsequent learning and may be a useful exercise particularly in situations where the teaching content is novel or unfamiliar to learners.

Implementing a teaching programme also requires a consideration of the attitudes of learners. From a constructivist perspective in the healthcare professional training, knowledge, skills and attitudes are acquired in the process of active learning [38]. Knowledge is the condition of being aware of facts and concepts which are the foundation for the ability to apply the skills to perform a task or to modify an attitude. Attitude is a way of thinking or feeling about objects, people and situations and is reflected in a person’s behaviour. Changes in attitude will bring about changes in people’s behaviour. Understanding Chinese doctors’ attitudes and beliefs towards communication skills will have fundamental importance for training program designers and teachers. GPs reported that they ignored the importance of, and were reluctant to focus on (attitude), the use of communication skills (behaviour) and were lack of opportunities to learn and acquire information (knowledge). This highlights that the objectives of communication skills training programs should not only be knowledge and skills-based but also include more attitudinal objectives. GPs in China need such a change of attitude in order for them to apply communication skills. This change in attitude ned time and culture change, and also structural changes in the system.

To our knowledge, this is the first study using NGT to identify communication skills training priorities and relevant issues not only for Chinese GPs but other countries health providers in diabetes care. This may inform a high-quality evidence-based training programs to support diabetes care improvement in primary care. We suggested that communication skills training in diabetes is important and how this can be implemented in the absence of statement in current Chinese diabetes guidelines [7]. We looked how GPs thought and what really matters to people with diabetes in their communication in the context of transforming primary healthcare system in China [39]. The methodology used in our study can also be seen as a paradigm of evidence-based GP training program formation for chronic conditions in China where 400,000 new GPs will be trained in the next 10 years [40].

There are several limitations to the NGT study. First, it may possible that participants in the NGT group may not be familiar with the pre-defined communication skills item. We did not collect the information on participants’ communication skill training backgrounds although GPs reported that they rarely received such training in previous focus group study. They may have risked misunderstanding the items, which could have influenced their responses. However, an information pack were sent out one week prior to the NGT group and participants made ratings in the first round. In addition, the facilitator of the NGT group briefly described the listed communication skills item at the start of the NGT group. Each of these approaches could improve participants’ understanding of items. Second, the sample was drawn from a single city in China. Although Guangzhou is a modern industrial city with close to fifteen million urban residents and about 5000 GPs, there still might be some difference in prioritization of outcomes in other geographic regions with different cultures in China. However, similar methodology could be adopted to find their local training priorities. Third, in our NGT groups we only include GPs rather than people with diabetes to prioritize communication skills. There might be a missing out on the collaboration with people with diabetes. However, when consider it as an educational program for GPs (learner-centered), their views on training feasibility were first investigated. In the next stage, there could be a pilot study to assess preliminary effects of training Chinese GPs in communication skills in diabetes care. Diabetes patients’ experience and other patient-important outcomes in diabetes management will then be evaluated. Fourth, we used online focus group rather than in-field discussion which may lead to less face-to-face discussion. However, our participants were familiar with such online methods and facilitators were trained, and protocol was followed to ensure the NGT group discussion quality. Although we identified the core themes in communication skills training for Chinese general practitioners in diabetes care, we still need to test it in a training program in future research. Our study prioritised ‘importance’ and ‘feasibility’; factors that had been highlighted through previous research with GP’s and patients with diabetes. However, we recognise that different areas of focus may be required in different geographical areas, or at different stages of training curriculum development. It may be necessary to revisit an NGT approach in the implementation stage of training, embedding an ongoing consideration of learner and patient perspective. Feedback from GPs, as well as patients, will be also important for modifying training content. We believe this could be a way to improve future diabetes management in primary care in China.

## Conclusion

Designing a training programme for communication skills in China may represent a paradigm shift for learners, and the literature has indicated that a ‘one size fits all’ approach to programme design across different environments should be undertaken with caution. Our programme of research aimed to understand the perspectives of patients and GP’s and built on these findings to identify priorities for communication skills training for Chinese GPs in diabetes care. A particular area of concern in our context was the constraints faced by GPs to implement quality communication in their care of diabetes patients. By designing a training programme based on elements of communication that are both important and feasible to learners, we would suggest that there is scope for enhanced engagement of GPs, which offers the potential for improved patient outcomes for patients with diabetes.

## Supplementary Information

**Additional file 1:** 

**Additional file 2:** 

**Additional file 3:** 

## Data Availability

The data that support the findings of this study are available from First Affiliated Hospital of Sun Yat-sen University but restrictions apply to the availability of these data, which were used under license for the current study, and so are not publicly available. Data are however available from the corresponding author (Professor Wei Chen) upon reasonable request and with permission of First Affiliated Hospital of Sun Yat-sen University.

## References

[1] Saeedi P, Petersohn I, Salpea P, Malanda B, Karuranga S, Unwin N, Colagiuri S, Guariguata L, Motala AA, Ogurtsova K et al: Global and regional diabetes prevalence estimates for 2019 and projections for 2030 and 2045: Results from the international diabetes federation diabetes atlas, 9th edition. Diabetes Research and Clinical Practice 2019, 157:107843. 10.1016/j.diabres.2019.107843 31518657

[2] Buse JB, Ginsberg HN, Bakris GL, Clark NG, Costa F, Eckel R, Fonseca V, Gerstein HC, Grundy S, Nesto RW (2007). Primary prevention of cardiovascular diseases in people with diabetes mellitus. Diabetes Care.

[3] Tuttle KR, Bakris GL, Bilous RW, Chiang JL, de Boer IH, Goldstein-Fuchs J, Hirsch IB, Kalantar-Zadeh K, Narva AS, Navaneethan SD (2014). Diabetic kidney disease: a report from an ADA consensus conference. Diabetes Care.

[4] Wang L, Gao P, Zhang M, Huang Z, Zhang D, Deng Q, Li Y, Zhao Z, Qin X, Jin D (2017). Prevalence and Ethnic Pattern of Diabetes and Prediabetes in China in 2013. JAMA.

[5] China NHCotPsRo: China health statistical yearbook 2019. Beijing: Peking union medical college publishing house; 2019.

[6] Ji L, Hu D, Pan C, Weng J, Huo Y, Ma C, Mu Y, Hao C, Ji Q, Ran X (2013). Primacy of the 3B approach to control risk factors for cardiovascular disease in type 2 diabetes patients. Am J Med.

[7] Jia W, Weng J, Zhu D, Ji L, Lu J, Zhou Z, Zou D, Guo L, Ji Q, Chen L (2019). Standards of medical care for type 2 diabetes in China 2019. Diabetes Metab Res Rev.

[8] Zolnierek KB, Dimatteo MR (2009). Physician communication and patient adherence to treatment: a meta-analysis. Med Care.

[9] Naik AD, Kallen MA, Walder A, Street RL (2008). Improving hypertension control in diabetes mellitus: the effects of collaborative and proactive health communication. Circulation.

[10] Levinson W, Pizzo PA: Patient-physician communication: it's about time. JAMA 2011(305(17)):1802–1803. 10.1001/jama.2011.556 21540424

[11] Margaret Lloyd RB (2018). Lorraine Noble: Clinical Communication Skills for Medicine.

[12] Wang JWLY, Li LX (2021). Diabetes management in China: types and reflections. Chinese General Practice.

[13] Jia W, Tong N (2015). Diabetes prevention and continuing health-care reform in China. Lancet Diabetes Endocrinol.

[14] Li YFWHX, Wang JJ (2020). Study of the interventions of contracted service model for community general practice team on multiple chronic diseases. Chinese Community Doctors.

[15] Chinese Diabetes Society NOFPD (2018). National guidelines for the prevention and control of diabetes in primary care. Chinese Journal of Internal Medicine.

[16] Renders CM, Valk GD, Griffin SJ, Wagner EH, van Eijk JT, Assendelft WJJ (2001). Interventions to improve the management of diabetes in primary care, outpatient, and community settings: a systematic review. Diabetes Care.

[17] Pearson-Stuttard J, Bennett J, Cheng YJ, Vamos EP, Cross AJ, Ezzati M, Gregg EW (2021). Trends in predominant causes of death in individuals with and without diabetes in England from 2001 to 2018: an epidemiological analysis of linked primary care records. Lancet Diabetes Endocrinol.

[18] Li X, Lu J, Hu S, Cheng KK, De Maeseneer J, Meng Q, Mossialos E, Xu DR, Yip W, Zhang H (2017). The primary health-care system in China. Lancet (London, England).

[19] Murphy F (2018). China's plan for 500 000 new GPs. BMJ.

[20] Liu X, Rohrer W, Luo A, Fang Z, He TH, Xie W (2015). Doctor-patient communication skills training in mainland China: a systematic review of the literature. Patient Educ Couns.

[21] Yao M (2022). Zhang D-y, Fan J-t, Lin K, Haroon S, Jackson D, Li H, Chen W, Cheng KK, Lehman R: The experiences of people with type 2 diabetes in communicating with general practitioners in China – a primary care focus group study. BMC Primary Care.

[22] Yao M (2021). Zhang D-y, Fan J-t, Lin K, Haroon S, Jackson D, Li H, Chen W, Lehman R, Cheng KK: The experiences of Chinese general practitioners in communicating with people with type 2 diabetes—a focus group study. BMC Fam Pract.

[23] Yao M (2021). Zhou X-y, Xu Z-j, Lehman R, Haroon S, Jackson D, Cheng KK: The impact of training healthcare professionals’ communication skills on the clinical care of diabetes and hypertension: a systematic review and meta-analysis. BMC Fam Pract.

[24] Skivington K, Matthews L, Simpson SA, Craig P, Baird J, Blazeby JM, Boyd KA, Craig N, French DP, McIntosh E (2021). A new framework for developing and evaluating complex interventions: update of medical research council guidance. BMJ.

[25] Sendall MC, McCosker LK, Brodie A, Hill M, Crane P (2018). Participatory action research, mixed methods, and research teams: learning from philosophically juxtaposed methodologies for optimal research outcomes. BMC Med Res Methodol.

[26] Mukhalalati BA, Taylor A (2019). Adult learning theories in context: a quick guide for healthcare professional educators. J Med Educ Curric Dev.

[27] Raine R, Sanderson C, Hutchings A, Carter S, Larkin K, Black N (2004). An experimental study of determinants of group judgments in clinical guideline development. Lancet (London, England).

[28] Cantrill JA, Sibbald B, Buetow S (1996). The Delphi and nominal group techniques in health services research. Int J Pharm Pract.

[29] McMillan SS, King M, Tully MP (2016). How to use the nominal group and Delphi techniques. Int J Clin Pharm.

[30] O'Cathain A, Murphy E, Nicholl J (2008). The quality of mixed methods studies in health services research. J Health Serv Res Policy.

[31] Notice on the issuance of Guangzhou General Practitioners (Family Doctors) Training Program (2019–2021) [http://www.gz.gov.cn/zwgk/zdgzlsqk/2019nzdgz/qjqmsbzhshzlcx/content/post_2868427.html]

[32] Population size and distribution in Guangzhou in 2019 [http://tjj.gz.gov.cn/tjdt/content/post_5727607.html]

[33] List of hospital institutions and community health care service centres in Guangzhou [http://wjw.gz.gov.cn/fwcx/yljgcx/content/post_7153475.html]

[34] Gazmararian JA, Williams MV, Peel J, Baker DW (2003). Health literacy and knowledge of chronic disease. Patient Educ Couns.

[35] Schillinger D, Grumbach K, Piette J, Wang F, Osmond D, Daher C, Palacios J, Sullivan GD, Bindman AB (2002). Association of health literacy with diabetes outcomes. JAMA.

[36] Golbeck AL, Ahlers-Schmidt CR, Paschal AM, Dismuke SE (2005). A definition and operational framework for health numeracy. Am J Prev Med.

[37] Cavanaugh K, Huizinga MM, Wallston KA, Gebretsadik T, Shintani A, Davis D, Gregory RP, Fuchs L, Malone R, Cherrington A (2008). Association of numeracy and diabetes control. Ann Intern Med.

[38] Cleland JA, Durning SJ, Artino AR: Researching medical education Chichester, England : Wiley Blackwell, 2015.; 2015.

[39] Li X, Krumholz HM, Yip W, Cheng KK, De Maeseneer J, Meng Q, Mossialos E, Li C, Lu J, Su M (2020). Quality of primary health care in China: challenges and recommendations. The Lancet.

[40] Opinions of the General Office of the State Council on Reforming and Improving General Practitioner Training and Incentive Mechanisms [http://www.gov.cn/zhengce/content/2018-01/24/content_5260073.html]

