# Supplementary material for: Using a mixed method to identify communication skills training priorities for Chinese general practitioners in diabetes care
Source: BMC Prim Care. 2022 Oct 15;23:262. doi: 10.1186/s12875-022-01868-8 (PMC9569069; doi:10.1186/s12875-022-01868-8)
Supplement: Supplementary file 1 — Additional file 1: [file 12875_2022_1868_MOESM1_ESM.docx]

**Good Reporting of A Mixed Methods Study (GRAMMS) checklist.**

O'Cathain A, Murphy E, Nicholl J. The quality of mixed methods studies in health services research. J Health Serv Res Policy. 2008;13: 92-98.

1. **Describe the justification for using a mixed methods approach to the research question**

As communication skills training is a complex intervention with multiple components, the MRC conceptual framework was considered to encourage good quality. Developing training programs for GPs is an educational activity that involves interaction between designers, educators, and learners. For researcher involvement in the process, we used the methods of action research. GPs had their medicine degrees from universities or colleges, qualifications in general practice and clinical practice experience in their workplace. We applied adult learning theories to understand their learning and practice behaviours, especially in a changing and reforming primary health care system context. With learning from those theories, a theoretical framework was developed to guide this mixed method research by a systematic and iterative approach to identify and refine communication skills training programs for GPs in managing diabetes patients (Figure 1).


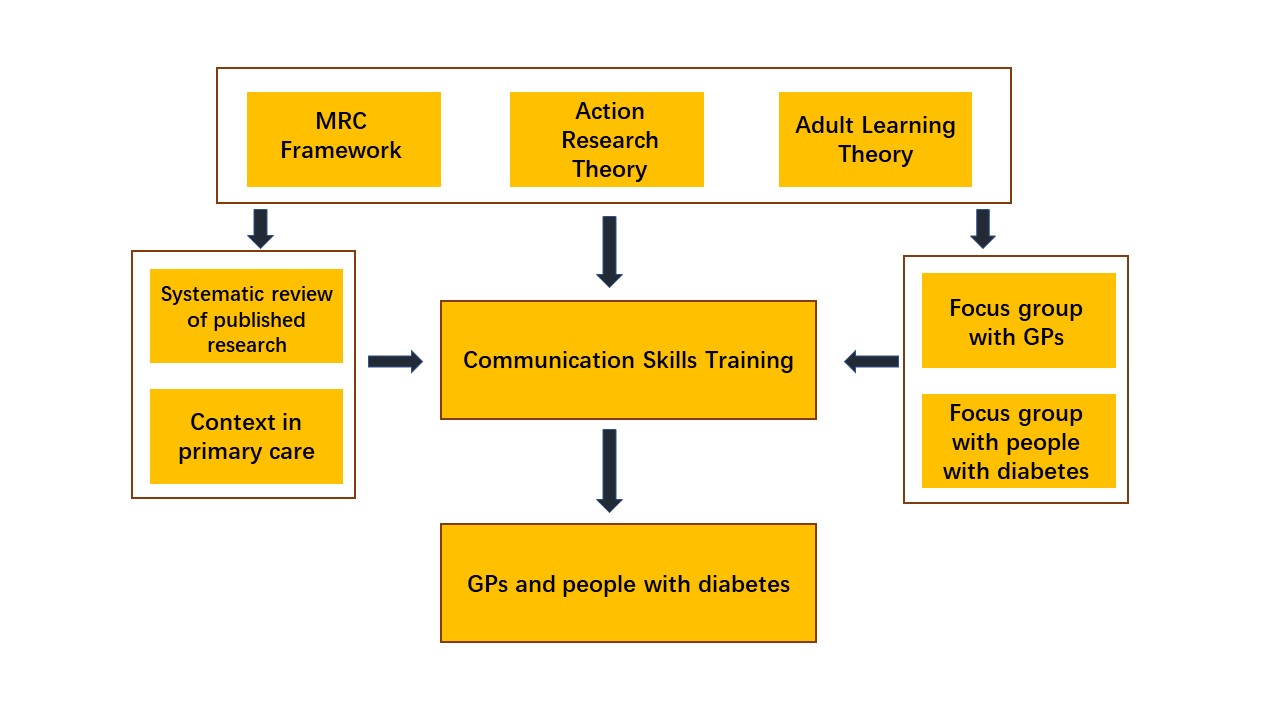


1. **Describe the design in terms of the purpose, priority and sequence of methods**

A systematic review was conducted to find existing evidence in communication skills training in diabetes care worldwide. Two focus group studies were then conducted with GPs and people with diabetes in China. The results of above studies have been described in detail elsewhere. Data from the above studies were combined to inform potential communication skills components for training. In this article, we specifically reported the details of using nominal group technique (NGT) to evaluate, refine and rate these components.

1. **Describe each method in terms of sampling, data collection and analysis**

We recruited GPs from a Guangzhou GP training program which began 2019 and is supported by the Guangzhou Municipal Health Commission. This training program mainly focuses on the improvement of GPs’ clinical skills, with the help of specialists from hospitals in Guangzhou. Purposeful sampling was used to select 60 GPs in this program from 30 community health service centers in 11 districts (both urban and suburban) throughout Guangzhou city. All GPs were qualified in general practice and had more than 3 years of work experience as required by the program.

The participants then independently rated each item immediately at the end of the group discussion, using an online survey. The qualitative data of group discussions was collected by video recording of the focus groups and field notes were made by facilitators.

All quantitative data were analyzed using STATA 16, including participants’ demographic characteristics and the Likert ratings of importance and feasibility of the training components. We calculated the mean Likert score ratings for all participants. For qualitative data, two of the researchers (MY and XJ) initially reviewed the entire transcripts of the 8 NGT group discussions. Transcripts were imported into NVivo12 software and coded independently by two researchers (MY and GY). Data were analyzed inductively by thematic analysis based on the principles of grounded theory.

1. **Describe where integration has occurred, how it has occurred and who has participated in it**

Based on the systematic review of the literature, and qualitative research with patients and GPs, a provisional list of communication skills training priorities was developed by researchers (MY & GY). The systematic review identified several communication skills training contents used from previous trials and showed impact on diabetes care. The qualitative studies illuminated the importance of context in implementing communications skills training, especially the socioeconomic and health care system background in China. These items, along with their descriptions, are outlined below in **Table 1.** We critically reviewed and analysed the evidence from the academic literature and provided refinement on terminology and descriptions for each of the identified training components.

1. **Describe any limitation of one method associated with the present of the other method**

There are several limitations to the NGT study. First, it may possible that participants in the NGT group may not be familiar with the pre-defined communication skills item. They may have risked misunderstanding the items, which could have influenced their responses. However, an information pack were sent out one week prior to the NGT group and participants made ratings in the first round. In addition, the facilitator of the NGT group briefly described the listed communication skills item at the start of the NGT group. Each of these approaches could improve participants’ understanding of items. Another limitation is that the NGT study was conducted online rather than in-person due to a small outbreak of Covid-19 during that time in Guangzhou. However, facilitators in NGT groups were trained before and a pilot NGT group was tested with protocols, which ensured the quality of NGT. Third, in our NGT groups we only include GPs rather than people with diabetes to prioritize communication skills. There might be a missing out on the collaboration with people with diabetes. However, when consider it as an educational program for GPs (learner-centered), their views on training feasibility were first investigated. In the next stage, there could be a pilot study to assess preliminary effects of training Chinese GPs in communication skills in diabetes care. Diabetes patients’ experience and other patient-important outcomes in diabetes management will then be evaluated.

1. **Describe any insights gained from mixing or integrating methods**

Details were described in the discussion. Several reasons affected the prioritization from the qualitative analysis of NGT groups. Although most GPs tended to believe that using various a variety of communication skills in medical encounters can promote diabetes patients’ better understanding of diabetes and improve diabetes management, several factors arising from doctors themselves, their patients and the external environment impede their potential needs from being met in training. They acknowledged that different aspects of communications skills were too burdensome or complex to implement. For some skills, such as motivational interviewing and shared decision making, most of GPs had no conception and had not even heard of them until they participated in this study. They also had no previous training experience in terms of a lot of communication skills, which may have made them choose more familiar items when rating. The reasons why GPs prioritize the top 3 of communication skills as health education, discuss blood glucose monitoring and explanation, and diabetes complications and CVD risk communication might be that they were able to acquire relevant knowledge with relative ease. And when learning or using such skills, they had guidelines for reference which were relatively objective, clear, and explicit. But although GPs discussed that it was hardly realistic to adopt time consuming or multistep communication skills, such as exploring diabetes patients’ emotional and psychosocial problems, and evaluating patients’ confidence, those skills still had a relative high ratings and rankings.
