# Supplementary material for: Using a mixed method to identify communication skills training priorities for Chinese general practitioners in diabetes care
Source: BMC Prim Care. 2022 Oct 15;23:262. doi: 10.1186/s12875-022-01868-8 (PMC9569069; doi:10.1186/s12875-022-01868-8)
Supplement: Supplementary file 2 — Additional file 2: [file 12875_2022_1868_MOESM2_ESM.docx]

**Supplementary Table 1 Details of GP participants in 8 NGT workshops: gender, age, education background, professional title, and location of practices in Guangzhou^a^ (n=58)**

|  | Group 1  (N=8; M3, F5)  Facilitators:  MY & LL | Group 2  (N=7; M6, F1)  Facilitators:  KL & BL | Group 3  (N=7; M1, F6)  Facilitators:  GY & HT | Group 4  (N=7; M3, F4)  Facilitators:  RW & JX | Group 5  (N=8; M4, F4)  Facilitators:  LL & GY | Group 6  (N=7; M4, F3)  Facilitators:  HT & LB | Group 7  (N=7; M4, F3)  Facilitators:  MY & JX | Group 8  (N=7; M4, F3)  Facilitators:  KL & RW |
| --- | --- | --- | --- | --- | --- | --- | --- | --- |
| GP 1 | 43, 16, E3, P3, D1 | 37, 14, E2, P2, D2 | 33, 8, E2, P2, D2 | 35, 10, E2, P2, D1 | 32, 6, E3, P2, D1 | 45, 20, E2, P2, D1 | 38, 16, E1, P2, D1 | 38, 12, E2, P3, D2 |
| GP 2 | 44, 22, E2, P2, D1 | 37, 8, E1, P2, D2 | 45, 22, E2, P3, D2 | 37, 13, E2, P2, D2 | 36, 12, E2, P2, D1 | 38, 15, E2, P1, D2 | 46, 26, E2, P2, D1 | 36, 11, E2, P3, D2 |
| GP 3 | 40, 16, E2, P3, D1 | 42, 22, E2, P1, D2 | 39, 9, E2, P2, D2 | 35, 7, E2, P2, D1 | 49, 25, E2, P4, D1 | 38, 14, E2, P2, D1 | 35, 11, E2, P2, D1 | 43, 20, E2, P4, D1 |
| GP 4 | 39, 10, E2, P3, D1 | 40, 15, E2, P3, D2 | 43, 19, E2, P3, D2 | 34, 12, E2, P2, D2 | 36, 9, E2, P2, D1 | 40, 11, E2, P2, D1 | 36, 6, E2, P2, D1 | 32, 2, E2, P1, D1 |
| GP 5 | 39, 8, E3, P3, D1 | 37, 2, E2, P2, D2 | 42, 14, E2, P3, D2 | 34, 7, E3, P2, D1 | 51, 10, E2, P4, D1 | 39, 5, E2, P2, D1 | 37, 13, E2, P3, D1 | 38, 3, E2, P2, D1 |
| GP 6 | 38, 7, E2, P2, D1 | 35, 5, E2, P2, D2 | 33, 18, E2, P2, D2 | 31, 4, E3, P2, D1 | 35, 11, E2, P2, D1 | 41, 17, E2, P3, D1 | 41, 15, E2, P2, D1 | 35, 11, E3, P2, D1 |
| GP 7 | 40, 15, E2, P2, D1 | 37, 11, E2, P2, D2 | 35, 10, E2, P2, D2 | 38, 14, E3, P3, D1 | 35, 7, E2, P1, D1 | 31, 5, E2, P2, D1 | 43, 21, E2, P3, D2 | 49, 16, E2, P3, D2 |
| GP 8 | 36, 12, E2, P2, D1 |  |  |  | 49, 24, E2, P3, D1 |  |  |  |

^a^ Gender (M/F), Age (years), GP experience (years worked as GPs), Education background (E1-E3, E1 College degree, E2 Bachelor’s degree, E3 Master’s degree), Professional title (P1-P4, P1 physician, P2 attending physician, P3 associate chief physician, P4 chief physician), District in Guangzhou (D1-D2, D1 city center, D2 rural or suburb)

Note: MY is both a male doctor and PhD student; LL, HT are female doctors and GP trainers; KL, BL, GY, RW are male doctors and GP trainers; JX is a female nurse and researcher. All facilitators were trained.
