# Supplementary material for: Using a mixed method to identify communication skills training priorities for Chinese general practitioners in diabetes care
Source: BMC Prim Care. 2022 Oct 15;23:262. doi: 10.1186/s12875-022-01868-8 (PMC9569069; doi:10.1186/s12875-022-01868-8)
Supplement: Supplementary file 3 — Additional file 3: [file 12875_2022_1868_MOESM3_ESM.docx]

Supplementary Table 2 A. Last round score and ranking of 19 communication skills items by GPs in the NGT groups: feasibility

| **Items** | **Group 1** | | **Group 2** | | **Group 3** | | **Group 4** | | **Group 5** | | **Group 6** | | **Group 7** | | **Group 8** | | **Total** | |
| --- | --- | --- | --- | --- | --- | --- | --- | --- | --- | --- | --- | --- | --- | --- | --- | --- | --- | --- |
|  | **Score** | **Rank** | **Score** | **Rank** | **Score** | **Rank** | **Score** | **Rank** | **Score** | **Rank** | **Score** | **Rank** | **Score** | **Rank** | **Score** | **Rank** | **Score** | **Rank** |
| **1 Active listening** | 57 | 6 | 51 | 1 | 44 | 8 | 48 | 8 | 64 | 2 | 41 | 10 | 49 | 4 | 54 | 1 | 408 | 5 |
| **2 Express empathy** | 58 | 5 | 35 | 11 | 46 | 6 | 50 | 6 | 64 | 2 | 47 | 6 | 46 | 7 | 48 | 5 | 394 | 6 |
| **3 Share bad news** | 52 | 10 | 33 | 12 | 45 | 7 | 42 | 13 | 61 | 5 | 44 | 9 | 46 | 7 | 45 | 7 | 368 | 13 |
| **4 Use examples** | 63 | 3 | 45 | 5 | 51 | 2 | 55 | 2 | 64 | 2 | 55 | 1 | 53 | 2 | 52 | 2 | 438 | 2 |
| **5 Idea, concerns and expectations** | 55 | 8 | 33 | 12 | 46 | 6 | 46 | 10 | 56 | 9 | 41 | 10 | 44 | 9 | 39 | 11 | 360 | 14 |
| **6 Nonverbal skills** | 52 | 10 | 38 | 10 | 51 | 2 | 44 | 11 | 53 | 11 | 35 | 13 | 45 | 8 | 39 | 11 | 357 | 15 |
| **7 Negotiation of behavioral change** | 56 | 7 | 44 | 6 | 41 | 9 | 47 | 9 | 63 | 3 | 49 | 5 | 43 | 10 | 44 | 8 | 387 | 9 |
| **8 Evaluate the patients’ confidence, support patients’ self-efficacy and optimism** | 47 | 12 | 31 | 14 | 47 | 5 | 43 | 12 | 52 | 12 | 29 | 15 | 44 | 9 | 39 | 11 | 332 | 17 |
| **9 Motivational interviewing** | 44 | 13 | 32 | 13 | 45 | 7 | 36 | 16 | 47 | 14 | 24 | 16 | 48 | 5 | 36 | 12 | 312 | 18 |
| **10 Shared decision making** | 55 | 8 | 41 | 9 | 48 | 4 | 54 | 3 | 59 | 7 | 46 | 7 | 44 | 9 | 43 | 9 | 390 | 8 |
| **11 Discuss blood glucose monitoring and explanation** | 66 | 1 | 49 | 3 | 51 | 2 | 53 | 4 | 62 | 4 | 50 | 4 | 55 | 1 | 47 | 6 | 433 | 3 |
| **12 Diabetes complications and cardiovascular disease risk communication** | 60 | 4 | 47 | 4 | 50 | 3 | 49 | 7 | 60 | 6 | 45 | 8 | 52 | 3 | 50 | 3 | 413 | 4 |
| **13 Medication adherences** | 53 | 9 | 50 | 2 | 46 | 6 | 51 | 5 | 53 | 11 | 40 | 11 | 44 | 9 | 48 | 5 | 385 | 10 |
| **14 Follow up or referring** | 60 | 4 | 50 | 2 | 45 | 7 | 40 | 14 | 58 | 8 | 40 | 11 | 49 | 4 | 43 | 9 | 385 | 11 |
| **15 Cultural biases and patients background awareness** | 55 | 8 | 43 | 7 | 51 | 2 | 46 | 10 | 55 | 10 | 38 | 12 | 47 | 6 | 43 | 9 | 378 | 12 |
| **16 Explore the patient's emotional and psychosocial (mental health) problems** | 47 | 12 | 29 | 15 | 37 | 10 | 37 | 15 | 43 | 16 | 33 | 14 | 38 | 11 | 35 | 13 | 299 | 9 |
| **17 Use online or telephone communication technic** | 50 | 11 | 32 | 13 | 46 | 6 | 35 | 17 | 46 | 15 | 41 | 10 | 44 | 9 | 42 | 10 | 336 | 16 |
| **18 Health education** | 64 | 2 | 51 | 1 | 52 | 1 | 56 | 1 | 67 | 1 | 53 | 2 | 53 | 2 | 49 | 4 | 445 | 1 |
| **19 Patient held health record management** | 56 | 7 | 42 | 8 | 46 | 6 | 48 | 8 | 50 | 13 | 52 | 3 | 52 | 3 | 47 | 6 | 393 | 7 |

Supplementary Table 2 B. Last round score and ranking of 19 communication skills items by GPs in the NGT groups: the importance

| **Items** | **Group 1** | | **Group 2** | | **Group 3** | | **Group 4** | | **Group 5** | | **Group 6** | | **Group 7** | | **Group 8** | | **Total** | |
| --- | --- | --- | --- | --- | --- | --- | --- | --- | --- | --- | --- | --- | --- | --- | --- | --- | --- | --- |
|  | **Score** | **Rank** | **Score** | **Rank** | **Score** | **Rank** | **Score** | **Rank** | **Score** | **Rank** | **Score** | **Rank** | **Score** | **Rank** | **Score** | **Rank** | **Score** | **Rank** |
| **1 Active listening** | 69 | 1 | 62 | 2 | 58 | 5 | 60 | 1 | 67 | 2 | 58 | 4 | 49 | 10 | 57 | 1 | 480 | 7 |
| **2 Express empathy** | 69 | 1 | 61 | 3 | 55 | 8 | 56 | 5 | 64 | 4 | 57 | 5 | 55 | 6 | 53 | 3 | 470 | 9 |
| **3 Share bad news** | 68 | 2 | 57 | 7 | 56 | 7 | 57 | 4 | 63 | 5 | 57 | 5 | 56 | 5 | 54 | 2 | 468 | 10 |
| **4 Use examples** | 65 | 5 | 58 | 6 | 57 | 6 | 60 | 1 | 60 | 6 | 60 | 2 | 55 | 6 | 51 | 4 | 466 | 11 |
| **5 Idea, concerns and expectations** | 66 | 4 | 60 | 4 | 62 | 2 | 60 | 1 | 68 | 1 | 60 | 2 | 57 | 4 | 47 | 8 | 480 | 8 |
| **6 Nonverbal skills** | 68 | 2 | 59 | 5 | 58 | 5 | 52 | 8 | 60 | 6 | 54 | 8 | 51 | 8 | 47 | 8 | 449 | 16 |
| **7 Negotiation of behavioral change** | 68 | 2 | 62 | 2 | 61 | 3 | 59 | 2 | 68 | 1 | 61 | 1 | 56 | 5 | 50 | 5 | 485 | 2 |
| **8 Evaluate the patients’ confidence, support patients’ self-efficacy and optimism** | 60 | 8 | 59 | 5 | 60 | 4 | 57 | 4 | 63 | 5 | 55 | 7 | 56 | 5 | 47 | 8 | 457 | 14 |
| **9 Motivational interviewing** | 62 | 6 | 58 | 6 | 56 | 7 | 54 | 7 | 58 | 8 | 47 | 11 | 58 | 3 | 49 | 6 | 442 | 17 |
| **10 Shared decision making** | 67 | 3 | 63 | 1 | 63 | 1 | 58 | 3 | 67 | 2 | 57 | 5 | 57 | 4 | 51 | 4 | 483 | 4 |
| **11 Discuss blood glucose monitoring and explanation** | 67 | 3 | 63 | 1 | 61 | 3 | 60 | 1 | 64 | 4 | 61 | 1 | 57 | 4 | 49 | 6 | 482 | 6 |
| **12 Diabetes complications and cardiovascular disease risk communication** | 67 | 3 | 62 | 2 | 60 | 4 | 60 | 1 | 65 | 3 | 59 | 3 | 59 | 2 | 53 | 3 | 485 | 3 |
| **13 Medication adherences** | 68 | 2 | 62 | 2 | 62 | 2 | 58 | 3 | 68 | 1 | 60 | 2 | 56 | 5 | 49 | 6 | 483 | 5 |
| **14 Follow up or referring** | 61 | 7 | 60 | 4 | 60 | 4 | 57 | 4 | 63 | 5 | 58 | 4 | 52 | 7 | 50 | 5 | 461 | 13 |
| **15 Cultural biases and patients background awareness** | 62 | 6 | 57 | 7 | 53 | 9 | 52 | 8 | 59 | 7 | 48 | 10 | 43 | 11 | 47 | 8 | 421 | 18 |
| **16 Explore the patient's emotional and psychosocial (mental health) problems** | 62 | 6 | 59 | 5 | 60 | 4 | 55 | 6 | 63 | 5 | 56 | 6 | 52 | 7 | 48 | 7 | 455 | 15 |
| **17 Use online or telephone communication technic** | 54 | 9 | 53 | 8 | 50 | 10 | 47 | 9 | 55 | 9 | 51 | 9 | 52 | 7 | 48 | 7 | 410 | 19 |
| **18 Health education** | 68 | 2 | 63 | 1 | 61 | 3 | 59 | 2 | 65 | 3 | 58 | 4 | 60 | 1 | 53 | 3 | 487 | 1 |
| **19 Patient held health record management** | 65 | 5 | 59 | 5 | 58 | 5 | 56 | 5 | 64 | 4 | 58 | 4 | 50 | 9 | 53 | 3 | 463 | 12 |
